# Associations between birthweight, gestational age at birth and subsequent type 1 diabetes in children under 12: a retrospective cohort study in England, 1998–2012

**DOI:** 10.1007/s00125-017-4493-y

**Published:** 2017-11-11

**Authors:** Raphael R. Goldacre

**Affiliations:** Unit of Health-Care Epidemiology, Big Data Institute, Li Ka Shing Centre for Health Information and Discovery, Nuffield Department of Population Health, University of Oxford, Oxford, OX3 7LF UK

**Keywords:** Birthweight, England/epidemiology, Gestational age, Hospital Episode Statistics, Large for gestational age, National Health Service, Preterm, Record linkage, Small for gestational age, Type 1 diabetes mellitus

## Abstract

**Aims/hypothesis:**

With genetics thought to explain only 40–50% of the total risk of type 1 diabetes, environmental risk factors in early life have been proposed. Previous findings from studies of type 1 diabetes incidence by birthweight and gestational age at birth have been inconsistent. This study aimed to investigate the relationships between birthweight, gestational age at birth and subsequent type 1 diabetes in England.

**Methods:**

Data were obtained from a population-based database comprising linked mother–infant pairs using English national Hospital Episode Statistics from 1998 to 2012. In total, 3,834,405 children, categorised by birthweight and gestational age at birth, were followed up through record linkage to compare their incidence of type 1 diabetes through calculation of multivariable-adjusted HRs.

**Results:**

Out of 3,834,405 children, 2969 had a subsequent hospital diagnosis of type 1 diabetes in childhood. Children born preterm (<37 weeks) or early term (37–38 weeks) experienced significantly higher incidence of type 1 diabetes than full term children (39–40 weeks) (HR 1.19 [95% CI 1.03, 1.38] and 1.27 [95% CI 1.16, 1.39], respectively). Children born at higher than average birthweight (3500–3999 g or 4000–5499 g) after controlling for gestational age experienced higher incidence of type 1 diabetes than children born at medium birthweight (3000–3499 g) (HR 1.13 [95% CI 1.03, 1.23] and 1.16 [95% CI 1.02, 1.31], respectively), while children at low birthweight (<2500 g) experienced lower incidence (0.81 [95% CI 0.67, 0.98]), signifying a statistically significant trend (*p* trend 0.001).

**Conclusions/interpretation:**

High birthweight for gestational age and low gestational age at birth are both independently associated with subsequent type 1 diabetes. These findings help contextualise the debate about the potential role of gestational and early life environmental risk factors in the pathogenesis of type 1 diabetes, including the potential roles of insulin sensitivity and gut microbiota.

**Electronic supplementary material:**

The online version of this article (10.1007/s00125-017-4493-y) contains peer-reviewed but unedited supplementary material, which is available to authorised users.

## Introduction

With the incidence of type 1 diabetes rising [1] and genetics thought to explain only 30–40% of total susceptibility [2], there is a need to define the supposed environmental factors that lead to seroconversion to positivity for diabetes-related autoantibodies in genetically susceptible individuals [3]. Gestational and early life risk factors have long been proposed [4, 5]. In 2001 there was a call for large studies to be conducted into whether birthweight is associated with type 1 diabetes [6]. Over 15 years later, such large studies are still lacking and this is still a controversial subject, with both high and low birthweight implicated [7]. While previous meta-analyses have shown positive associations between high birthweight and type 1 diabetes in overall pooled estimates, this finding has not been consistently demonstrated in all studies [8, 9]. Associations with low birthweight have been even less consistent as findings have varied considerably by study design [8, 9]. Any study of associations between birthweight and type 1 diabetes needs to consider that birthweight increases with gestational age at birth, and it has been argued that size for gestational age measures should be preferred to birthweight thresholds when assessing the relationship between birthweight and type 1 diabetes [10]. Preterm birth has itself been shown to be associated with type 1 diabetes in a meta-analysis [11]. Given the natural association between birthweight and gestational age, these findings may appear to be contradictory. The task of reconciling these various findings is made more difficult by the fact that previous meta-analyses in this area have been hindered by differing study populations and a lack of consistent adjustment for important confounders. There are also gaps in the literature; for example, few studies have investigated associations between early term or post-term birth and type 1 diabetes [12]. To tease out these relationships, while accounting for other potential confounding factors, a large dataset is required. The use of linked routinely collected hospital admissions and deaths data to study mother–infant pairs across time has been described previously [13], but few studies of this kind have been conducted on a national scale in England. The aim of this study was to determine whether birthweight, gestational age at birth and birthweight for gestational age (BFGA) are significantly associated with type 1 diabetes in childhood using a population-based dataset of routinely collected national statistics in England.

## Methods

### Data sources

This study used three record-linked data sources: (1) English national Hospital Episode Statistics (HES)—admitted patient care data for the whole of England, 1998–2012 [14]. These hospital record abstracts, routinely collected by the English national Health and Social Care Information Centre, contain details of every episode of admitted patient care (including day case care) occurring in English National Health Service (NHS) hospitals and NHS commissioned care in the private sector. The details in each record include date of admission and discharge, demographic information about the patient and the reasons for their admission to hospital, which include clinical diagnoses coded using the ICD (www.who.int/classifications/icd/en/). The English national HES first became linkable in 1998, with the collection of anonymised encrypted personal data items, and the most recent HES data provided by the national data provider to the Unit of Health-Care Epidemiology, Oxford University, was for 2012; (2) Maternity Hospital Episode Statistics (MHES) for the whole of England, 1998–2012 [15]. These are a subset of HES and are intended to cover every birth occurring in an NHS hospital or under NHS provision (including home deliveries). For each birth, there is a maternity record for the mother and a delivery record for the child. These are similar to regular HES records but, in addition to the usual information contained in HES, they include extra ‘tails’ of data that provide information about the mother’s characteristics during delivery and the child’s characteristics at birth. The maternity/delivery data items collected are described in detail in the HES Data Dictionary [16]; (3) National death registration data, 1998–2012. Death certification data are collected in England by the Office for National Statistics (ONS). Each death registration record contains demographic information about each deceased individual, date of death and diagnostic information about the cause of death, again coded using the ICD.

These three data resources have been linked together into a multipurpose mother–infant database at the Unit of Health-Care Epidemiology (UHCE), University of Oxford, such that each infant’s MHES record is linked to his or her successive records of hospitalisation and/or death in later life, as well as to the mother’s MHES record and her successive records of prior or subsequent hospitalisation and/or death. The UHCE has longstanding experience of linking routinely collected hospital admissions data to study individuals across time, specifically with the use of linked HES since its introduction and ONS death registration data, methods for which have been documented extensively elsewhere [17, 18]. The linkage of each infant’s birth record to its subsequent hospitalisation records (and any death record used in censoring on follow-up) was conducted by matching encrypted personal identifiers, which included HES-ID [19], NHS number (unique to each individual in England), date of birth and postcode. The mother–infant matching was achieved similarly using a mixture of deterministic and probabilistic methods (further information is provided in the electronic supplementary material [ESM] Methods).

The data resources were obtained for permitted use in this study and ethics approval was obtained from the Central and South Bristol Multi-Centre Research Ethics Committee (04/Q2006/176) for analysis of the record-linked data. Full access to the database was available for use in this study.

### Study design and population

**Fig. 1:**
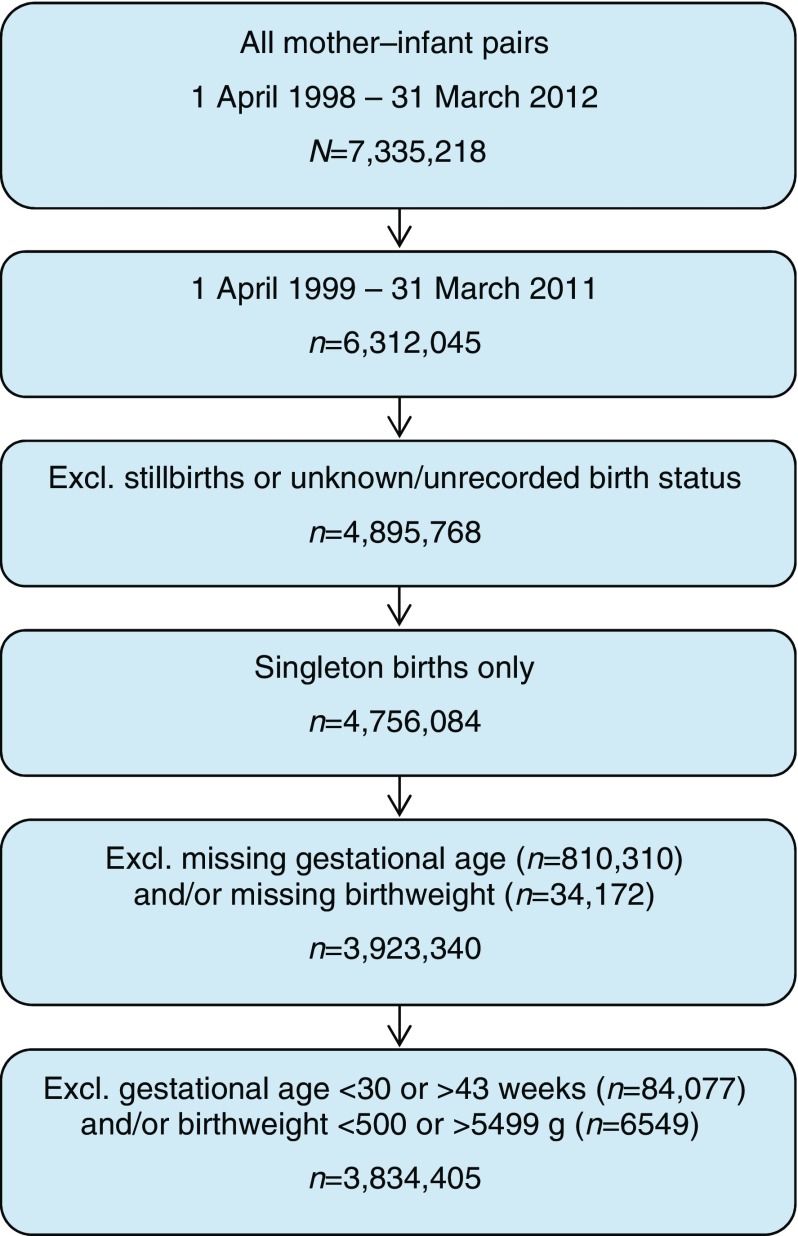
Flowchart showing the derivation of the 3,834,405 mother–infant pairs used in the analysis after exclusions (excl.)

In total, 7,335,218 mother–infant pairs were identified through mother–infant linkage of MHES records from 1 April 1998 to 31 March 2012. These pairs were extracted from the database, along with any other HES and/or death records belonging to either the mother or the child that occurred during the same period. The ESM Table shows the number of linked pairs by financial year, referenced to birth registry data from the ONS (all references to years are financial years such that 1998 means 1 April 1998 to 31 March 1999). The linked data were analysed using a retrospective cohort study design to compare the rates of type 1 diabetes in children by birthweight, gestational age at birth and BFGA. Children born in 1998 were excluded from the analysis to allow sufficient prior history for a diagnosis of gestational diabetes to be recorded, and children born in 2011 were excluded to allow each child at least 1 year of follow-up. After restricting to live births only, the number of mother–infant pairs was reduced to 4,895,768 (97% of those excluded had unknown or unrecorded birth status). Multiples were excluded because their fetal growth patterns are known to be atypical. Children with missing values for either birthweight or gestational age at birth were excluded. Children with a recorded birthweight <500 g or >5499 g and/or gestational age at birth <30 weeks or >43 weeks were excluded because of implausibility/non-viability and because previous validation studies of MHES have revealed these values to be commonly erroneous [20]. These exclusions (see Fig. 1) brought the total number of mother–infant pairs to 3,834,405.

### Exposure variables

#### Birthweight (grams)

The most recent meta-analysis [8] of the association between birthweight and type 1 diabetes grouped children according to the following birthweight categories: <2500 g, 2500–2999 g, 3000–3499 g, 3500–3999 g and ≥4000 g, with 3000–3499 g taken as the reference category. The same approach was taken in the present study; although a flexible approach to grouping was also adopted to explore the relationship.

#### Gestational age at birth (completed weeks)

The most recent meta-analysis [11] of the association between preterm birth and type 1 diabetes defined preterm birth as less than 37 completed weeks of gestation (i.e. <259 days). This is also the internationally accepted definition of preterm (ICD10 P07.3). Post-term pregnancy is internationally defined as pregnancy that has extended to or beyond 42 completed weeks of gestation (294 days) (ICD-10 P08.2). The definition of ‘term’ is debated but the American College of Obstetricians and Gynecologists Committee on Obstetric Practice Society for Maternal-Fetal Medicine recommend the following classifications [21]: preterm, <37 0/7 weeks; early term, 37 0/7 weeks through 38 6/7 weeks; full term, 39 0/7 weeks through 40 6/7 weeks; late term, 41 0/7 weeks through 41 6/7 weeks; and post-term, 42 0/7 weeks and beyond. The same groupings were used in the present study with ‘full term’ taken as the reference group.

#### BFGA

In previous studies BFGA has been measured in quintiles [10]. The same approach was taken in the present study. For each week of gestational age at birth, the children were grouped into quintiles of birthweight, so that each individual was coded between 1 and 5 with equal numbers of children in each quintile for each gestational week. This was done for male and female children separately, since boys are generally heavier for their gestational age than girls. A composite variable was then created, which brought together all of the data for the children in quintile 1, all of the data for the children in quintile 2 and so on. The same approach was taken to generate BFGA in deciles. While BFGA is a convenient way of summarising the effect of birthweight while simultaneously adjusting for gestational age at birth and sex, BFGA was not considered a substitute for looking at actual birthweight adjusted for gestational age in multivariable analyses.

Potential confounders or effect modifiers included maternal age in years (grouped <25, 25–29, 30–34, 35–39, >40); maternal type 1 diabetes (ICD-10 codes E10 or O24.0); maternal obesity (E66); gestational diabetes (O24.4 or O24.9); infant sex; area deprivation based on the mother’s Index of Multiple Deprivation (IMD) rank (in quintiles); and Caesarean section (elective and emergency combined).

### Follow-up and outcome measurement

Type 1 diabetes diagnoses were identified by searching each child’s subsequent HES records for ICD-10 diagnosis code E10 after the age of 9 months. Type 1 diabetes diagnosis before 9 months is extremely rare and any recorded type 1 diabetes diagnoses at this age, although coded as such, would almost certainly represent neonatal diabetes (ICD-10 P70.2) [2]. Date of entry to the study population for each infant was the 15 day of the month of their delivery discharge record (exact date of birth was not available from HES in compliance with data governance requirements). Since follow-up for all participants was measured from month of birth, cumulative follow-up time for each individual was approximately equivalent to age. Date of exit for each individual was the date of their earliest type 1 diabetes diagnosis record, if it occurred, otherwise date of death, if it occurred, otherwise the end of the follow-up period (31 March 2012).

### Statistical analysis

The crude incidence rate (per 100,000 years) of type 1 diabetes was calculated for each category of birthweight, gestational age at birth, and BFGA in quintiles and deciles. Mantel–Haenszel adjusted rate ratios were calculated to control for each of the secondary independent variables in turn, and adjusted HRs were calculated using Cox’s proportional hazards models to compare the groups after multivariable adjustment. Where appropriate, trend tests across exposure groups were conducted by entering the categorical variables into the models as continuous terms and using the likelihood ratio test (LRT) to check that model fit was not compromised. The proportional hazards assumption was tested formally by splitting age–time at 4.5 years so that there were equal numbers of outcomes in each age–time period and then testing for interactions between the primary exposure variables and age–time.

The strategy for building the Cox models was based on which other secondary independent variables had the strongest effect on the relationships between the exposure variables and type 1 diabetes (except for infant sex, which was considered an a priori confounder). Missing values were always dealt with in multivariable analyses by ensuring that any two models being compared contained the same observations.

All analyses were performed using Stata/IC 13.1 for Windows, StataCorp, TX, USA.

## Results

### Overview

Table 1 displays the known characteristics of the 3,834,405 mother–infant pairs who entered the analysis. The mean (± SD) birthweight was 3370 (±494 g). The median gestational age at birth was 40 weeks (interquartile range [IQR] 39–40). The median length of follow-up was 5.7 years (IQR 2.9–9.6). Out of 3,834,405 children born, 2969 were first diagnosed with type 1 diabetes at least 9 months after birth. The mean age at type 1 diabetes diagnosis after 9 months was 5.1 ± 2.9 years.

**Table 1:** Distribution of characteristics of the mother–infant pairs in the dataset

Exposure variable	*n*	%
Birthweight (g)		
<2500	203,846	5.3
2500–2999	642,599	16.8
3000–3499	1,411,152	36.8
3500–3999	1,138,447	29.7
4000–5499	438,361	11.4
Total	3,834,405	100
Missing	0	
Gestational age at birth (weeks)		
<37	213,799	5.6
37–38	738,451	19.3
39–40	1,952,998	50.9
41	761,960	19.9
42–43	167,197	4.4
Total	3,834,405	100
Missing	0	
Maternal age at delivery (years)		
<25	995,238	26.0
25–29	1,040,349	27.1
30–34	1,083,414	28.3
35–39	582,444	15.2
>40	131,798	3.4
Total	3,833,243	100
Missing	1162	
Maternal gestational diabetes^a^		
No	3,788,299	98.8
Yes	46,106	1.2
Total	3,834,405	100
Missing	0	
Maternal type 1 diabetes^a^		
No	3,758,249	98.0
Yes	76,156	2.0
Total	3,834,405	100
Missing	0	
Maternal obesity^a^		
No	3,740,398	97.5
Yes	94,007	2.5
Total	3,834,405	100
Missing	0	
Infant sex		
Male	1,961,175	51.2
Female	1,867,440	48.8
Total	3,828,615	100
Missing	5790	
Maternal IMD rank quintile		
1 (most deprived)	763,373	20.0
2	762,752	20.0
3	762,863	20.0
4	763,393	20.0
5 (least deprived)	763,832	20.0
Total	3,816,213	100
Missing	18,192	
Caesarean section		
No	2,966,896	77.4
Yes	867,509	22.6
Total	3,834,405	100
Missing	0	

^a^Based on known hospital records

### Crude rates analysis

In total, 2969 children received a hospital diagnosis of type 1 diabetes over an aggregate of 24,101,378 person-years, an overall rate of 12.3 per 100,000 person-years. The crude rates of type 1 diabetes by each category of each variable are shown in Table 2.

**Table 2:** Crude rates (per 100,000 person-years) of type 1 diabetes incidence within each stratum of each potential risk factor

Potential risk factor	Type 1 diabetes (*n*)	Rate	Rate ratio (95% CI)	LRT	*p* value
Birthweight (g)				5.9 (4)	0.2058
<2500	156	12.0	1.01 (0.86, 1.20)		
2500–2999	483	11.9	1.00 (0.90, 1.12)		
3000–3499	1051	11.9	Reference		
3500–3999	916	12.9	1.09 (0.99, 1.19)		
4000–5499	363	13.2	1.12 (0.99, 1.26)		
Gestational age (weeks)				56.4 (4)	<0.0001
<37	204	14.9	1.31 (1.13, 1.51)		
37–38	726	15.3	1.34 (1.23, 1.47)		
39–40	1382	11.4	Reference		
41	520	10.8	0.95 (0.86, 1.05)		
42–43	137	13.0	1.14 (0.96, 1.36)		
BFGA quintile				31.4 (1)	<0.0001, trend
1 (lightest)	510	10.4	Reference		
2	549	11.3	1.08 (0.96, 1.22)		
3	599	12.5	1.20 (1.07, 1.35)		
4	659	13.8	1.32 (1.18, 1.48)		
5 (heaviest)	651	13.7	1.31 (1.17, 1.47)		
BFGA decile				33.2 (1)	<0.0001, trend
1 (lightest)	239	9.7	Reference		
2	271	11.2	1.16 (0.97, 1.38)		
3	283	11.5	1.19 (1.00, 1.41)		
4	266	11.1	1.15 (0.97, 1.37)		
5	323	13.4	1.38 (1.17, 1.63)		
6	276	11.6	1.20 (1.01, 1.43)		
7	322	13.4	1.39 (1.17, 1.64)		
8	337	14.2	1.46 (1.24, 1.73)		
9	290	12.2	1.26 (1.06, 1.49)		
10 (heaviest)	361	15.2	1.57 (1.33, 1.84)		
Mother’s age at delivery (years)				12.7 (4)	0.0129
<25	704	11.2	Reference		
25–29	805	12.3	1.10 (0.99, 1.22)		
30–34	882	12.7	1.13 (1.03, 1.25)		
35–39	475	13.4	1.19 (1.06, 1.34)		
40–49	103	14.3	1.28 (1.04, 1.57)		
Maternal gestational diabetes^a^				3.6 (1)	0.0594
No	2910	12.3	Reference		
Yes	59	15.9	1.3 (1.00, 1.68)		
Maternal type 1 diabetes^a^				203.4 (1)	<0.0001
No	2880	12.0	Reference		
Yes	89	90.6	7.55 (6.12, 9.33)		
Maternal obesity^a^				1.9 (1)	0.1625
No	2897	12.3	Reference		
Yes	72	14.6	1.19 (0.94, 1.5)		
Infant sex				0.2 (1)	0.6446
Male	1532	12.4	Reference		
Female	1436	12.2	0.98 (0.92, 1.06)		
IMD rank quintile				5.3 (4)	0.256
1 (most deprived)	549	11.5	Reference		
2	567	12.1	1.05 (0.93, 1.18)		
3	590	12.4	1.08 (0.96, 1.21)		
4	628	13.0	1.13 (1.01, 1.27)		
5 (least deprived)	627	12.7	1.1 (0.98, 1.24)		
Caesarean section				20.2 (1)	<0.0001
No	2209	11.8	Reference		
Yes	760	14.3	1.21 (1.12, 1.32)		

Crude rate ratios denoting, for each potential risk factor, the ratio of type 1 diabetes incidence compared with the reference group indicated

^a^Based on known hospital records

LRTs and *p* values indicate the overall variability within each variable (or trend where indicated)

Absolute birthweight was not associated with type 1 diabetes, but BFGA was strongly associated as the rate of type 1 diabetes increased significantly with BFGA (Table 2). Children born preterm (<37 weeks) and early term (37–38 weeks) were significantly more likely to be diagnosed with type 1 diabetes compared with children born at 39 or 40 weeks (Table 2). Higher gestational age was not significantly associated with type 1 diabetes in the crude or multivariable analyses.

### Multivariable analysis

#### Gestational age

In Cox regression models, after controlling for infant sex and for the apparent confounding effects of mother’s type 1 diabetes status, children born preterm (adjusted HR 1.19 [95% CI 1.03, 1.38]) or early term (adjusted HR 1.27 [95% CI 1.16, 1.39]) experienced significantly higher incidence of type 1 diabetes than full term children (Table 3). Further adjustment for other covariates made no material difference to these results. No significant association was found between late term or post-term birth and subsequent type 1 diabetes.

**Table 3:** Adjusted Cox’s proportional hazard ratios comparing incidence of type 1 diabetes in cohorts of children born at different gestational ages

Gestational age (weeks)	Type 1 diabetes (*n*)	Partially adjusted^a^ HR (95% CI)	Further adjusted^b^ HR (95% CI)	Fully adjusted^c^ HR (95% CI)
Preterm: <37	204	1.29 (1.12, 1.50)	1.19 (1.03, 1.38)	1.17 (1.01, 1.36)
Early term: 37–38	726	1.33 (1.22, 1.46)	1.27 (1.16, 1.39)	1.24 (1.13, 1.36)
Full term: 39–40	1382	Reference	Reference	Reference
Late term: 41	520	0.95 (0.86, 1.05)	0.95 (0.86, 1.05)	0.95 (0.86, 1.06)
Post-term: 42–43	137	1.14 (0.95, 1.35)	1.14 (0.96, 1.36)	1.14 (0.96, 1.36)

^a^Adjusted for infant sex

^b^Adjusted for infant sex and maternal type 1 diabetes

^c^Adjusted for infant sex, maternal type 1 diabetes, gestational diabetes, maternal obesity, maternal age, deprivation quintile and Caesarean section

Covariates were included in the model categorised as presented in Table 1

#### Birthweight

As in the analysis of crude rates, birthweight unadjusted for gestational age was not significantly associated with type 1 diabetes incidence. After controlling for infant sex and a modest confounding effect of maternal type 1 diabetes, increased birthweight adjusted for gestational age was significantly associated with increased type 1 diabetes incidence (Table 4). Children born at 3500–3999 g and 4000–5499 g experienced a significantly higher incidence of type 1 diabetes than children born at 3000–3499 g, while children in the lowest birthweight category (<2500 g) experienced a significantly lower incidence. Re-categorising birthweight into ten groups split at 500 g intervals showed an 8% increase in risk per 500 g increase (HR 1.08 [1.04, 1.12]; *p* trend <0.001). Further adjustment for other factors did not materially alter the results and the general pattern of risk remained (*p* trend 0.01) (Table 4). There were no significant interactions between birthweight and gestational age at birth.

**Table 4:** Adjusted Cox’s proportional hazard ratios comparing incidence of type 1 diabetes in cohorts of children born at different birthweights, with and without adjustment for gestational age

Birthweight (g)	Type 1 diabetes (*n*)	Partially adjusted^a^ HR (95% CI)	Gestational-age adjusted^b^ HR (95% CI)	Further adjusted^c^ HR (95% CI)	Fully adjusted^d^ HR (95% CI)
<2500	156	1.01 (0.85, 1.19)	0.76 (0.63, 0.92)	0.81 (0.67, 0.98)	0.82 (0.67, 0.99)
2500–2999	483	1.00 (0.90, 1.11)	0.90 (0.81, 1.01)	0.92 (0.82, 1.03)	0.93 (0.83, 1.04)
3000–3499	1051	Reference	Reference	Reference	Reference
3500–3999	916	1.09 (0.99, 1.19)	1.15 (1.05, 1.25)	1.13 (1.03, 1.23)	1.11 (1.02, 1.22)
4000–5499	363	1.12 (0.99, 1.26)	1.20 (1.07, 1.36)	1.16 (1.02, 1.31)	1.12 (0.99, 1.27)

^a^Adjusted for infant sex

^b^Adjusted for infant sex and gestational age

^c^Adjusted for infant sex, gestational age and maternal type 1 diabetes

^d^Adjusted for infant sex, gestational age, maternal type 1 diabetes, gestational diabetes, maternal obesity, maternal age, deprivation quintile and Caesarean section

Covariates were included in the model categorised as presented in Table 1

#### BFGA

**Fig. 2:**
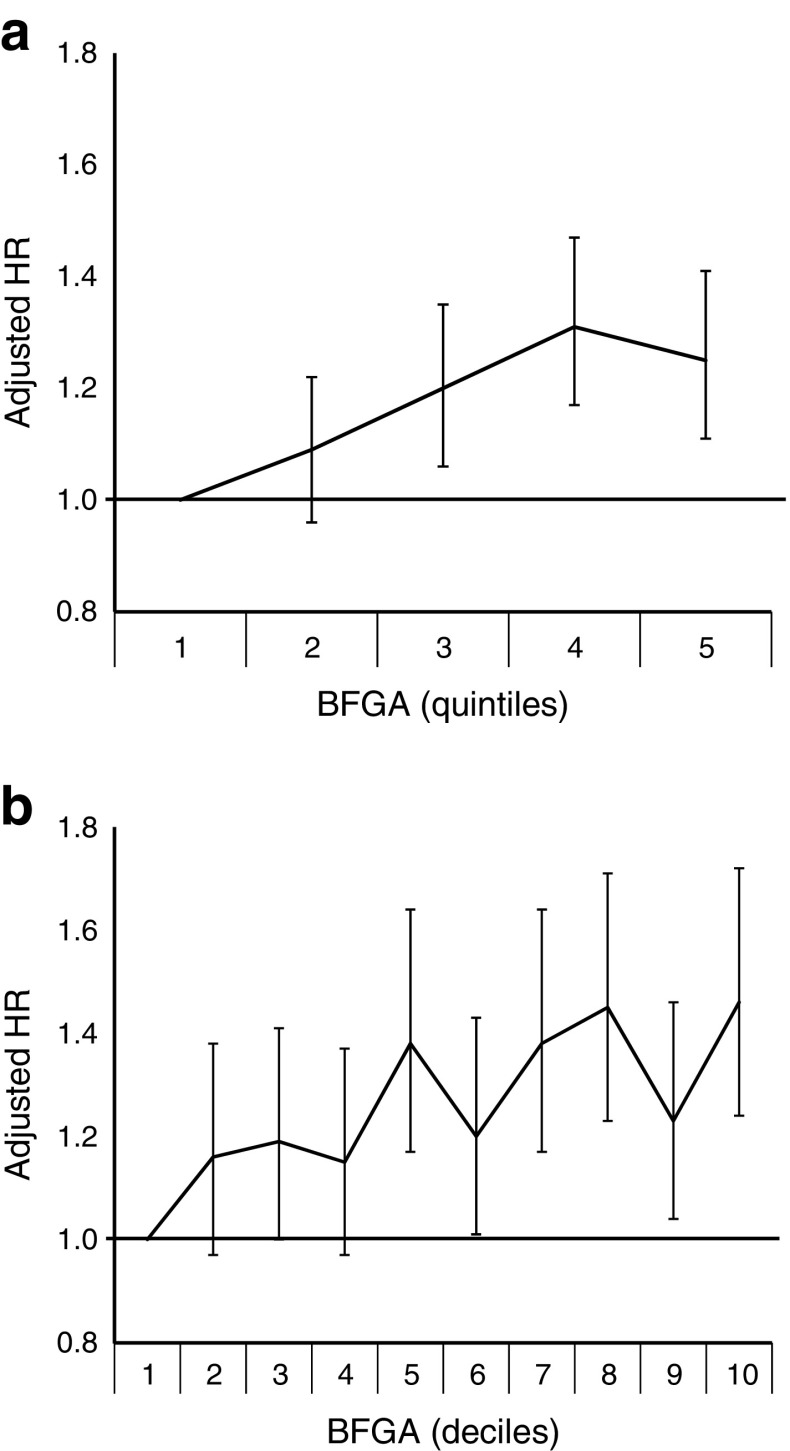
Adjusted Cox proportional hazard ratios comparing incidence of type 1 diabetes in cohorts of children by BFGA in (**a**) quintiles and (**b**) deciles. *p* trend <0.0001 for both models. Error bars are 95% CI. Note: infant sex is already accounted for in the calculation of BFGA. The HRs were further adjusted in the Cox model for maternal type 1 diabetes, gestational diabetes, maternal obesity, maternal age, deprivation quintile and Caesarean section. Covariates were included in the model categorised as presented in Table 1

The significant association between high BFGA and type 1 diabetes persisted after multivariable adjustment (Fig. 2). Entering BFGA in quintiles or deciles as continuous variables did not compromise the fit of the models and, for both quintiles and deciles, the trend across BFGA categories was highly statistically significant (*p* trend <0.0001).

### Sensitivity analyses

Exclusion from the dataset of children whose mother had type 1 diabetes and/or gestational diabetes made no material difference to the results. Restricting the study period to 2003–2010 (when linkage of mother–infant pairs was greater [ESM Table]) did not materially change the results. The HRs did not differ significantly by sex and the assumption of proportional hazards throughout follow-up was satisfied.

## Discussion

### Summary of principal findings

After controlling for various potential confounders, children born preterm (<37 weeks) and early term (37–38 weeks) experience an approximately 20–25% higher incidence of type 1 diabetes in early childhood than children born full term. Absolute birthweight, unadjusted for gestational age, is not significantly associated with type 1 diabetes incidence. However, high BFGA is significantly associated with an increased rate of type 1 diabetes and proves a useful measure for describing the adjusted birthweight–type 1 diabetes relationship. After adjusting for gestational age and other factors, children born with higher birthweight (3500–3999 g or ≥4000 g) are approximately 10–15% more likely than children of medium birthweight (3000–3499 g) to be diagnosed with type 1 diabetes. Furthermore, children in the lowest birthweight category (<2500 g) are significantly less likely to be diagnosed with type 1 diabetes, by about 20%.

### Comparison with previous literature

A meta-analysis of preterm birth (<37 weeks) and type 1 diabetes published in 2014 reported that preterm birth was associated with an 18% increased risk of type 1 diabetes (pooled OR 1.18 [95% CI 1.11, 1.25]) [11]. This pooled estimate is almost the same as that reported by a large single study from Sweden published in 2015 by Khashan et al [12]. The present finding is almost identical to both of these estimates. The meta-analysis did not investigate potential associations between late term or post-term birth and type 1 diabetes, while Khashan et al reported a low relative incidence of type 1 diabetes (adjusted rate ratio 0.87 [95% CI 0.83, 0.90]) in children born ≥41 weeks, which was moderated in nested sibling analysis with full adjustment. The findings from the sibling analysis by Khashan et al are consistent with those of the present study.

A meta-analysis of birthweight and type 1 diabetes published in 2010 reported that children with birthweight >4 kg had an increased risk of 10% compared with children weighing 3.0–3.5 kg at birth (pooled OR 1.10 [95% CI 1.04, 1.19]) [8]. This pooled estimate is adjusted for gestational age, which was reported not to have a strong confounding effect, unlike in the present study where unadjusted birthweight was not found to be associated with type 1 diabetes. Nevertheless, the adjusted pooled estimate from the meta-analysis is almost identical to the adjusted estimate from the current study. The present study also finds a significantly decreased rate of type 1 diabetes in children weighing <2500 g at birth. Although Cardwell et al [8] did not find the rate of type 1 diabetes to be decreased in this group when combining both cohort and case–control studies, the pooled odds ratio for cohort studies alone was 0.79 (95% CI 0.67, 0.92), which is very similar to the present finding. Furthermore, the pattern reported by Khashan et al [12] of an increasing rate of diabetes with increased BFGA is the same as in the present study and the effect sizes are compatible.

The sensitivity analysis suggests that the associations are similar in children diagnosed under 4.5 years and in those diagnosed between 4.5 and 12 years of age. However, the observed associations may not hold for adult-onset type 1 diabetes.

### Strengths and limitations

A considerable strength of this study is its large size, covering 13 years of prospectively collected record-linked data and comprising nearly four million pairs of mothers and children in an integrated national healthcare system. Such high statistical power enables risk stratification by several categories of birthweight and gestational age while controlling for multiple other factors in a single population. The fact that all data were collected longitudinally removes the possibility of recall bias and selection biases commonly associated with case–control studies. Notwithstanding issues of coverage (below), the accuracy of the data collected has generally been good and the data are well validated for birthweight in the range 500–5499 g and for gestational age in the range 30–43 weeks [20]. HES have been previously validated for type 1 diabetes using the Yorkshire Register of Diabetes in Children and Young People, which has an estimated ascertainment of 99% [22]. The case counts in HES and the Yorkshire register were very similar (2224 vs 2161) and, in person-based matching, 90.8% of hospital admissions in HES since 2000 were successfully matched to cases in the Yorkshire register. The study concluded that HES could successfully serve as a surrogate national diabetes register [22]. Even if the absolute rates reported in Table 2 are underestimates to the extent that non-hospitalised type 1 diabetes cases are not captured, the relative measures of incidence are valid measures of association provided that the shortfall is non-differential across the exposure groups.

However, there were limitations to the study. The extent of the linkage between mothers and children was lower for 1999–2002 than 2003–2010 (ESM Table). However, this is unlikely to have caused bias providing that the shortfall was random (notably, restricting the study population to 2003–2010 did not materially change the results) but it does reduce statistical power. Similarly, missing values for birth status, birthweight and gestational age meant that the number of mother–infant pairs used in the analysis was substantially reduced leading to loss of power since some hospitals are less thorough than others in supplying the full range of data items from the delivery episode [23], but this cause of missing data would be unlikely to affect the representativeness of the cohorts in terms of their risk of subsequent type 1 diabetes.

The dataset did not contain information on some important variables which could conceivably confound, modify or mediate the effects of birthweight and/or gestational age at birth. These include early infant feeding [24, 25], infection during gestation [26], susceptibility to infection during early childhood [4], mother’s BMI [27] and mother’s weight gain during gestation [28]. Genotype data were also unavailable in this study; however, previous studies have demonstrated no shared genetic link between birthweight and type 1 diabetes [29, 30]. The absence of a maternal gestational diabetes diagnosis in hospital does not necessarily mean that the mother did not have gestational diabetes during the pregnancy. In this dataset, 2% of children (76,156/3,834,405) were born to mothers with known gestational diabetes (Table 1). This is at the lower end of other prevalence estimates [31]. Similarly, only 2.5% of children were born to obese mothers, a clear underestimate. Given the rarity of type 1 diabetes, even a hypothetical under-reporting of maternal diabetes is unlikely to explain the associations. On the other hand, common factors such as obesity, overweight and weight gain during pregnancy are much more prevalent and could conceivably account for the present results if there are shared mechanisms [32].

#### Potential mechanisms

The effects of low gestational age and high BFGA appear somewhat contradictory. One hypothesis points to the role of insulin resistance in type 1 diabetes, also known as ‘double diabetes’ [33]. Preterm birth can lead to catch-up growth in early life which, in turn, can lead to reduced insulin sensitivity [34]. While the ‘catch-up’ hypothesis may seem attractive in light of the present finding of an association between preterm or early term birth and type 1 diabetes, the potential role of insulin resistance does not easily explain the pattern of increased risk of type 1 diabetes with increased BFGA, especially as birthweight has previously been shown to have an inverse relationship with type 2 diabetes [35]. On the other hand, many other studies have failed to find an association between small for gestational age and insulin sensitivity in later childhood [34], while among children born to mothers with type 1 diabetes large for gestational age has been found to be associated with neonatal glycaemic dysregulation [36]. To speculate, while preterm birth might be a predeterminant of early life factors that affect insulin resistance, BFGA could conceivably be on the pathway between nutritional intake and glycaemic regulation in the mother and altered beta cell function in the metabolic programming of the child [37–39]. A persuasive alternative explanation for the preterm birth effect is that gut dysbiosis, which is more common in preterm infants [40], may underlie the pathogenesis of type 1 diabetes [38, 41]. The association between gut dysbiosis and type 1 diabetes has gathered substantial momentum in recent research [42]. Further studies of the influence of gut microbiota on seroconversion to positivity for diabetes-related autoantibodies in children born at different gestational ages would help to elucidate these relationships.

## Conclusion

High BFGA and preterm or early term birth are independently associated with subsequent type 1 diabetes. As incidence of type 1 diabetes increases, birth cohort studies of type 1 diabetes and early life risk factors help to contextualise the debate about potential mechanisms of action in type 1 diabetes disease pathogenesis. These findings suggest that the still-undefined environmental factors that lead to seroconversion to positivity for diabetes-related autoantibodies in genetically susceptible individuals are rooted in gestation and early life. This study is also an example of what can be done using national routinely collected data to follow mother–infant pairs through record linkage.

## Electronic supplementary material

ESM (PDF 160 kb)

## Abbreviations

BFGA: Birthweight for gestational age
HES: Hospital Episode Statistics
IMD: Index of Multiple Deprivation
IQR: Interquartile range
LRT: Likelihood ratio test
MHES: Maternity Hospital Episode Statistics
NHS: National Health Service
ONS: Office for National Statistics
UHCE: Unit of Health-Care Epidemiology
