# Supplementary material for: Associations between birthweight, gestational age at birth and subsequent type 1 diabetes in children under 12: a retrospective cohort study in England, 1998–2012
Source: Diabetologia. 2017 Nov 11;61(3):616–25. doi: 10.1007/s00125-017-4493-y (PMC6448964; doi:10.1007/s00125-017-4493-y)
Supplement: Supplementary file 1 — (PDF 160 kb) [file 125_2017_4493_MOESM1_ESM.pdf]

ESM Methods

Matching mother-infant pairs in HES

Scope

This document describes the process of matching prepared mother and infant records identified from the national Hospital Episodes Statistics (HES) dataset for the purposes of this study. Reference is made to data fields described in the HES Inpatient Data Dictionary [1]. The background of NHS Maternity Statistics, and some discussion of the historical challenges regarding their use, have been more fully documented elsewhere [2-4].

Identifying delivery and birth records

There are a number of ways of identifying delivery and birth records in HES [4]. For this study, delivery and birth records were identified by the presence of a delivery/birth “tail” of data items attached to the HES record. These tails of data provide information about the mother’s characteristics at delivery and the child’s characteristics at birth. For any given birth episode, the values of every data field on the tail of the mother’s record should ideally match those on the baby’s record. In practice, this is not always the case, particularly as the tails can sometimes contain missing data for certain fields. The matching methods described below, therefore, required that some, but not all, of these values matched.

Matching methods

There is no gold standard for linkage of mother-infant pairs in HES [4]. The approach in this study was to be more concerned with ensuring that a match was correct (i.e. maximising true positives), and less concerned about throwing away uncertain matches. The rationale for taking this more restrictive approach was to reduce the possibility of bias, since incorrectly matched mother-infant pairs (i.e. the inclusion of false positives) would lead to a blurring of the true effects of baseline characteristics. The principal disadvantage of discarding uncertain matches in these circumstances is reduction of statistical power. Given that the numbers in this population-based study were already very high, a reduction in statistical power was regarded as an acceptable trade-off.

Matching mother-infant pairs was achieved using a mixture of deterministic and probabilistic methods. Deterministic matching was used to create provisional pairs, while probabilistic

matching was used to break provisional pairs, i.e. to identify the "best pair" in circumstances where deterministic methods caused a conflict, such as where one baby matched to more than one mother.

Deterministic matching

Deterministic links were identified as a pair of records (one belonging to a mother, the other belonging to a baby) agreeing exactly on baby's date of birth (which was obtained from "dob" on the stem of the infant's record and "dobbaby" on the tail of the mother's record), and two or more positive matches on the following fields: encrypted postcode, encrypted mother's date of birth ("dob" on the mother's record and "motdob" on the child's record), birthweight ("birweit"), local patient identifier ("lopatid"), provider code ("procode"), GP practice ("gpprac").

10,061,381 pairs from 1998 to 2012 passed this test and were kept and stored in a provisional matched pair output table and further used for match resolution using probabilistic matching.

Probabilistic matching

In order to compare one match pair with another, to identify the "best" pair where deterministic methods caused a conflict, competing pairs were scored by assigning weights to each match field. The additional fields used in probabilistic matching were hospital admission date ("admidate"), discharge date ("disdate"), place of delivery ("delplace"), delivery method ("delmeth"), purchaser code ("purcode"), resident local authority ("resladst"), mother's age ("age" on the mother's record and "matage" on the child's record), sex of infant ("sex" on the child's record and "sexbaby" on the mother's record), birth order ("birorder"), total number of babies ("numbab"), gestational age ("gestat"). These fields were used along with the deterministic matching fields above to corroborate a match, to identify multiple births, and to determine whether one match pair was better than another where the deterministic match fields created multiple matched pairs (of which only one pair was likely to be a true match).

Matching fields with more distinct values have more discrimination than those with fewer distinct values, and as such they were assigned a higher power score. Negative values were set for mismatches too. Competing pairs were sorted and then ranked to identify the highest scoring pair. A pair was accepted only if it had the highest score in both mother-to-baby matching (i.e. where the baby was the best match for the mother) *and* in baby-to-mother

matching (i.e. where the mother was the best match for the baby). Pairs that did not meet these criteria were discarded. After match resolution, there were 7,335,218 pairs from 1 April 1998 to 31 March 2012.

References:

1. NHS Health and Social Care Information Centre (2010) Hospital Episode Statistics: Inpatient Data Dictionary. Available from http://www.hscic.gov.uk/media/1358/HES-Hospital-Episode-Statistics-HES-Admitted-Patient-Data-Dictionary/pdf/HES_Inpatients_DD_Sept10.pdf, accessed 12 July 2017

2. NHS Health and Social Care Information Centre (2015). NHS Maternity Statistics - England, 2013-14. Available from http://www.hscic.gov.uk/catalogue/PUB16725, accessed 12 July 2017

3. Dattani N. Linking maternity data for England, 2005–06: methods and data quality. Health Statistics Quarterly, Spring 2011 (2012) Available from http://www.ons.gov.uk/ons/rel/hsq/health-statistics-quarterly/no--53--spring-2012/linkage-of-maternity-hospital-episode-statistics-data.html, accessed 12 July 2017

4. Harron K, Gilbert R, Cromwell D, van der Meulen J (2016). Linking Data for Mothers and Babies in De-Identified Electronic Health Data. PLoS One;11:e0164667

ESM Table

ESM Table: Annual number of matched mother-infant pairs in Maternity HES (MHES) referenced to the annual number of births in England as collected by the Office for National Statistics (ONS)

Financial year	ONS registered births in England	MHES linked mother-infant pairs	Ratio of linked MHES pairs to ONS registrations (%)
1998	598,950	378,879	63.3
1999	585,308	398,102	68.0
2000	570,556	394,355	69.1
2001	564,235	418,552	74.2
2002	571,745	475,793	83.2
2003	594,184	528,884	89.0
2004	608,645	540,710	88.8
2005	618,708	553,967	89.5
2006	640,650	571,723	89.2
2007	663,781	577,450	87.0
2008	667,932	598,686	89.6
2009	674,949	622,717	92.3
2010	682,892	631,106	92.4
2011	689,582	644,294	93.4
Total	8,737,052	7,335,218	84.0
